# Эндокринные нарушения у пациентов с трансфузионно-зависимыми наследственными анемиями

**DOI:** 10.14341/probl13149

**Published:** 2022-12-20

**Authors:** А. В. Витебская, Е. С. Бугакова, Е. А. Писарева, Ю. В. Тихонович

**Affiliations:** ФГАОУ ВО Первый Московский государственный медицинский университет имени И.М. Сеченова Министерства здравоохранения Российской Федерации (Сеченовский Университет); ФГАОУ ВО Первый Московский государственный медицинский университет имени И.М. Сеченова Министерства здравоохранения Российской Федерации (Сеченовский Университет); ФГАОУ ВО Первый Московский государственный медицинский университет имени И.М. Сеченова Министерства здравоохранения Российской Федерации (Сеченовский Университет); ФГАОУ ВО Первый Московский государственный медицинский университет имени И.М. Сеченова Министерства здравоохранения Российской Федерации (Сеченовский Университет)

**Keywords:** гипопитуитаризм, гипогонадизм, сахарный диабет, анемия Даймона–Блекфена, бета-талассемия

## Abstract

Частые трансфузии эритроцитарной массы у детей с наследственными анемиями приводят к перегрузке железом, что может вызывать эндокринные осложнения, такие как задержка роста, гипотиреоз, гипогонадизм и нарушения углеводного обмена.Клинический случай 1. У пациента с трансфузионно-зависимой (ТЗ) анемией Даймонда–Блекфена в 16,3 года выявлены нарушенная гликемия натощак, недостаточность соматотропного гормона (СТГ), гипогонадотропный гипогонадизм; начата терапия рекомбинантным гормоном роста (рГР). В 16,8 года диагностированы вторичный гипотиреоз, вторичный гипокортицизм, сахарный диабет. В 17,2 года непрерывный мониторинг гликемии (НМГ) выявил подъемы гликемии до 11,7 ммоль/л. Терапия рГР и эфирами тестостерона продолжена; левотироксин и кортеф отменены пациентом. В 17,9 года рост 163 см; данных за гипотиреоз и гипокортицизм нет; гликемия в пределах целевых значений.Клинический случай 2. Пациентка с ТЗ-бета-талассемией майор. В 11,5 года выявлена недостаточность СТГ; терапия рГР с 12,8 до 15,3 года. В 13,8 года диагностирована задержка полового созревания. В 15,0 года выявлена гипергликемия 7,2 ммоль/л; при поведении перорального глюкозотолерантного теста (ПГТТ) — норма; по данным НМГ — подъемы гликемии до 9,5 ммоль/л. В 16 лет рост 152 см; в связи с остановкой полового созревания назначена заместительная гормональная терапия.ЗАКЛЮЧЕНИЕ. У пациентов с наследственными ТЗ-анемиями диагностированы нарушения роста, полового созревания и углеводного обмена, что подтверждает необходимость регулярного эндокринологического обследования. Для выявления нарушений углеводного обмена рекомендуется исследование гликемии натощак, ПГТТ и НМГ; исследование гликированного гемоглобина нецелесообразно.

## Abstract (en)

Often transfusions red blood cells in patients with hereditary anemias lead to iron overload, that can cause endocrine complications, such as growth retardation, hypothyroidism, hypogonadism, and disorders of carbohydrate metabolism.Clinical case 1. A boy with transfusion-dependent (TD) Diamond-Blackfan anemia at 16.3 years presented with impaired fasting glucose, growth hormone (GH) deficiency, hypogonadotropic hypogonadism; GH therapy was initiated. At the age of 16.8 years old secondary hypothyroidism, secondary hypocorticism and diabetes mellitus were diagnosed. At 17.2 years continuous glucose monitoring (CGM) detected glucose elevations up to 11.7 mmol/l. Therapy with GH and testosterone ethers was continued; levothyroxine and cortef were stopped by patient. At 17.9 years height was 163 cm; no data supporting hypothyroidism nor hypocorticism; glycaemia within goal range.Clinical case 2. A girl with TD beta-thalassemia major at the age of 11.5 years presented with GH deficiency; GH therapy has been conducted from 12.8 to 15.3 years of age. At 13.8 years retardation of pubertal development was diagnosed. At 15.0 hyperglycemia 7.2 mmol/l was detected; normal results of oral glucose tolerance test (OGTT) were observed; glycemia elevations were up to 9.5 mmol/l according to CGM data. At 16.0 height was 152 cm; because of pubertal development arrest hormone replacement therapy was prescribed.CONCLUSION. Growth, pubertal and carbohydrate metabolism disorders were diagnosed in patients with TD hereditary anemias, that confirms the necessity of regularly endocrine investigation. To detect impairment of carbohydrate metabolism investigation of fasting blood glucose, OGTT, and CGM is recommended; glycated hemoglobin measurement is not considered reasonable.

## АКТУАЛЬНОСТЬ

Эндокринные нарушения могут развиваться как осложнения тяжелых соматических заболеваний, значительно снижая качество жизни пациентов. Частые гемотрансфузии у детей с наследственными анемиями приводят к перегрузке организма железом — вторичному гемохроматозу. Железо накапливается в различных органах, в том числе в железах внутренней секреции, вызывая нарушение их функции. Среди эндокринных осложнений описаны гипотиреоз, задержка роста, гипогонадизм, нарушения углеводного обмена и др. [1][2]. В клинических рекомендациях ISPAD (International Society for Pediatric and Adolescent Diabetes, Международное общество по детскому и подростковому диабету) гемохроматоз рассматривается как одна из причин развития СД [3].

## ОПИСАНИЕ СЛУЧАЯ

## Клинический случай 1. Пациент Е с анемией Даймонда-Блекфена

В связи с жалобами на низкие темпы роста пациент был направлен на эндокринологическое обследование в 14-летнем возрасте.

Анамнез

Ребенок от 2-й беременности, первых срочных, самостоятельных родов. При рождении масса тела 2800 г, рост 48 см. Психомоторное развитие по возрасту. Наследственный анамнез по эндокринным заболеваниям не отягощен, все родственники выше 165 см, близкородственный брак родители отрицают. Рост матери 170 см, отца — 175 см.

В возрасте 2 мес выявлена апластическая анемия Даймонда–Блекфена (АДБ). До 2 лет мальчик находился на ежемесячной заместительной терапии эритроцитарной массой, далее до 7 лет получал метилпреднизолон с полной медикаментозной компенсацией, с 7 лет вновь переведен на гемотрансфузии, отмечается высокая трансфузионная зависимость — ежемесячно получает заместительную терапию эритроцитарной массой.

В 10 лет выявлена тяжелая перегрузка железом печени (концентрация железа в паренхиме 15,64 мг/г). Весь период наблюдения с целью выведения избытка железа пациент регулярно получал деферазирокс в дозе 1000–1750 мг/сут (30–35 мг/кг).

При обследовании в 16 лет по данным МРТ выявлена крайняя степень перегрузки железом печени (концентрации железа в паренхиме 27,9 мг/г), умеренная — миокарда и гипофиза. При УЗИ брюшной полости отмечены признаки гепатоспленомегалии, диффузных изменений паренхимы печени и поджелудочной железы, внутрибрюшная лимфаденопатия. В биохимическом анализе крови — признаки перегрузки железом (ферритин 2620 мкг/л (N 15-200), гепатита (аланинаминотрансфераза (АЛТ) 121 Ед/л, аспартатаминотрансфераза (АСТ) 45 Ед/л (N до 40)). В связи с жалобами на прогрессирующую задержку роста направлен на эндокринологическое обследование.

Результаты физикального, лабораторного и инструментального исследования

В 14,2 года впервые осмотрен эндокринологом (табл.1, рис. 1): рост в пределах нормы (SDS роста -1,70), половое созревание допубертатное. Выявлено отставание костного возраста (КВ), допубертатные значения гонадотропинов и тестостерона; данных за гипотиреоз не получено; клинических признаков надпочечниковой недостаточности не выявлено, ИФР-1 в пределах возрастной нормы. Рекомендовано динамическое наблюдение.

При обследовании в детском эндокринологическом отделении в 16,3 года (табл.1, рис. 1) впервые выявлена задержка роста (SDS роста -2,61) при отсутствии признаков полового созревания (Таннер 1); сохранялось отставание КВ (12,5 года от хронологического. При лабораторном исследовании выявлена нарушенная гликемия натощак; нарушений секреции пролактина, гормонов щитовидной железы и надпочечников не отмечено.

**Table:** Таблица 1. Показатели физического развития и результаты лабораторных исследований пациента Е.Table 1. Indicators of physical development and laboratory results of patient E *4 мес терапии рекомбинантным гормоном роста (рГР), через месяц после 4-й инъекции препарата эфиров тестостерона.**9 мес терапии рГР, 3 мес левотироксин 50 мкг, непостоянный прием кортефа, 6 мес без препаратов эфиров тестостерона.

Возраст, лет	Рост, см (SDS)	Вес, кг (SDSимт)	Половой статус по Таннеру, объем тестикул	Костный возраст,годы	ТТГ,мкМЕ/мл	свТ4,пмоль/л	Кортизол,нмоль/л	Пролактин,мкМЕ/мл	ИФР-1,нг/мл (SDS)	ЛГ,мМЕ/мл	ФСГ,мМЕ/мл	Тестостероннмоль/л	Глюкоза плазмы натощак, ммоль/л
14,2	148,0 (-1,70)	38,0 (-0,69)	Р1G1, 2 мл	11,5	1,42	11,2	-	-	157,5 (-1,40)	1,53	1,25	0	-
16,3	155,0 (-2,61)	45,0 (0,63)	Р1G1, 3 мл	12,5	1,6	12,0	566	300	141 (-3,28)	0,8	0,43	0,26	5,9–6,3
16,8 *	158,0 (-2,37)	49,0 (-0,32)	Р2G2, 4 мл	13,1	1,5	6,0	<28	280	268 (-1,29)	0,6	<0,3	2,1	5,0
17,2 **	160,0 (-2,15)	49,0 (-0,58)	Р2G2, 4 мл	-	-	12,3	73	-	374 (-0,48)	0,4	<0,3	1,0	6,5

**Figure:**
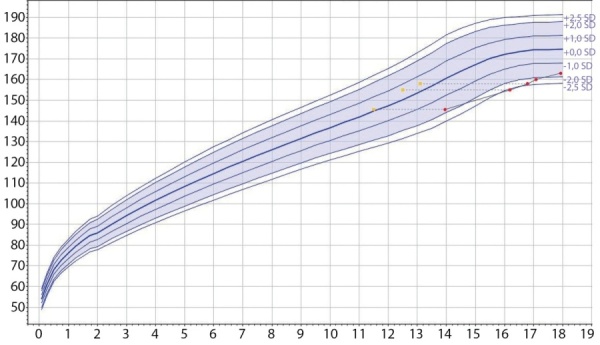
Рисунок 1. График роста пациента Е.Примечание к рисунку: по вертикали — рост, см; по горизонтали — возраст, годы; измерения роста в соответствии с хронологическим (красные точки) и костным (желтые точки) возрастом.Figure 1. Growth chart of patient E.

В связи с «нулевыми» значениями гонадотропинов при базальном исследовании был проведен тест с аналогом гонадотропин-рилизинг-гормона, получены допубертатные значения лютеинизирующего гормона (ЛГ) (табл. 2).

**Table:** Таблица 2. Тесты с аналогом гонадотропин-рилизинг-гормона пациента Е. в 16,3 и 16,8 годаTable 2. Tests with an analogue of gonadotropin-releasing hormone in patient E. at 16.3 and 16.8 years

Возраст	Гормоны	0 ч	1 ч	4 ч
16,3 лет	ЛГ, мМЕ/мл	0,4	2,5	3,7
ФСГ, мМЕ/мл	0,3	0,2	0,2
16,8 лет	ЛГ, мМЕ/мл	0,6	2,3	2,9
ФСГ, мМЕ/мл	<0,3	0,1	0,4

Учитывая задержку роста и низкий уровень инсулиноподобного ростового фактора 1-го типа (ИРФ-1; SDS=-3,28), для подтверждения недостаточности соматотропного гомона (СТГ) после предварительной подготовки препаратом эфиров тестостерона проведены две СТГ-стимуляционные пробы (с клофелином и инсулином). Получены низкие пиковые значения СТГ в двух тестах — 5,73 и 6,42 нг/мл соответственно (в норме выше 10 нг/мл, по крайней мере в одной из точек).

Таким образом, по результатам обследования выявлены недостаточность СТГ и нарушенная гликемия натощак вследствие вторичного гемохроматоза на фоне терапии трансфузиями изогруппной эритроцитарной массы по поводу АДБ. Результаты теста с аналогом гонадотропин-рилизинг-гормона не позволили исключить гипогонадотропный гипогонадизм.

С 16,5 года инициирована терапия рекомбинантным гормоном роста (рГР) в дозе 0,033 мг/кг. С целью инициации собственного полового созревания, а также дифференциальной диагностики гипогонадизма с задержкой полового созревания с 16,3до 16,7 года получил 4 инъекции препарата эфиров тестостерона в дозе 80 мг.

В 16,8 года (табл. 1, рис. 1) на фоне терапии отмечалось улучшение показателей роста (SDS роста -2,37), прогрессия полового развития (Таннер 2) и КВ. По данным лабораторного обследования отмечена нормализация ИФР-1 (SDS=-1,29), выявлены вторичный гипотиреоз, вторичный гипокортицизм. Назначена терапия левотироксином 50 мкг/сути, гидрокортизоном 7,5 мг/сут (5,1 мг/м2 площади поверхности тела).

В связи с выявлением гипергликемии натощак проведен пероральный глюкозотолерантный тест (ПГТТ) (табл. 3): выявлен сахарный диабет, однако, учитывая сохранную секрецию инсулина (38,2 мМЕ/мл), нормогликемию на фоне соблюдения диеты с ограничением углеводов с высоким гликемическим индексом, сахароснижающая терапия не была назначена, рекомендовано наблюдение в динамике.

**Table:** Таблица 3. Пероральный глюкозотолерантный тест пациента Е. в 16,8 годаTable 3. Oral glucose tolerance test of patient E. at 16.8 years

Возраст, лет	0 мин	60 мин	120 мин
Глюкоза, ммоль/л	6,8	10,8	11,9
Инсулин, мкМЕ/мл	38,2	86,5	247

В 17,2 лет (табл. 1, рис. 1) на фоне терапии рГР сохранялась тенденция к нормализации роста (SDS роста -2,15), половое созревание без динамики (Таннер 2). В гормональном статусе — нормальный уровень ИФР-1 (SDS=-0,48), эутиреоз на фоне заместительной терапии левотироксином, низкие значения кортизола и половых гормонов.

В течение 5 дней проводился непрерывный мониторинг гликемии (НМГ) (рис. 2), выявлено эпизодическое повышение постпрандиальной гликемии до 11,7 ммоль/л (211 мг/дл). Средний уровень гликемии составил 6,2±1,8 ммоль/л.

**Figure:**
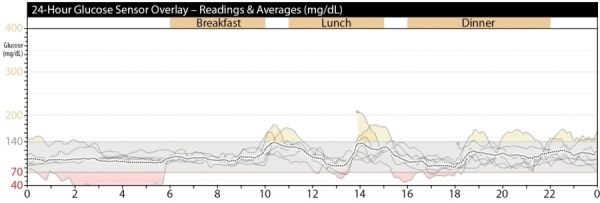
Рисунок 2. Результаты 5-дневного непрерывного мониторинга гликемии пациента Е.Примечание к рисунку: 200 мг/дл = 11,1 ммоль/л, 140 мг/дл = 7,8 ммоль/л, 70 мг/дл = 3,9 ммоль/л, 40 мг/дл = 2,2 ммоль/л.Figure 2. Results of 5-day continuous monitoring of glycemia of patient E.

Диагноз и тактика ведения

Гипопитуитаризм (на момент наблюдения недостаточность СТГ, вторичный гипотиреоз, вторичный гипокортицизм, гипогонадотропный гипогонадизм) манифестировал у пациента со вторичным гемохроматозом на фоне АДБ в 16 лет. На основании проведенного в 17 лет обследования был установлен диагноз сахарного диабета. По результатам мониторинга гликемии сахароснижающая терапия не показана. Рекомендовано соблюдение диеты с ограничением легкоусвояемых углеводов. Решено продолжить терапию рГР, левотироксином, кортефом, эфирами тестостерона; дозы препаратов скорректированы.

Исход

Пациент с гипопитуитаризмом и сахарным диабетом вследствие перегрузки железом на фоне трансфузионно-зависимой АДБ продолжил получать рГР и препарат эфиров тестостерона; самостоятельно отменил прием левотироксина и кортефа. При обследовании в детском эндокринологическом отделении в 17,9 года рост пациента составил 163 см (+8 см за 1,5 года терапии рГР); убедительных данных за гипотиреоз (свободный тироксин (св.Т4) 15,6 пмоль/л) и гипокортицизм (базальный кортизол 266 нмоль/л) не получено. Гликемия в пределах целевых значений.

## Клинический случай 2. Пациентка А. с бета-талассемией

Пациентка с жалобами на задержку роста в возрасте 11 лет была направлена на консультацию к эндокринологу гематологом.

Анамнез

Девочка от близкородственного брака, первой беременности, срочных самостоятельных родов. При рождении масса тела 2900 г, рост 49 см. Рост матери 160 см, отца — 165 см.

В 3 года диагностирована бета-талассемия майор (большая форма) (БТМ), проведена спленэктомия. Наблюдается гематологом, ежемесячно проводится заместительная терапия эритроцитарной массой. Согласно заключению гематолога, отмечается перегрузка железом (данные МРТ и ферритина не представлены). В течение всего периода наблюдения обращали на себя внимание типичные для гемохроматоза изменения: выраженная гиперпигментация кожи, пальпаторно и по данным УЗИ определяемое увеличение печени, повышение активности печеночных трансаминаз (при первом обращении АЛТ 102 Ед/л, АСТ 757 Ед/л (N до 40)). В описываемый период пациентка получала хелаторную терапию (деферазирокс в дозе 1000–1250 мг/сут (20–40 мг/кг/сут)), гепатопротекторы и препараты урсодезоксихолевой кислоты.

Результаты физикального, лабораторного и инструментального исследования

При первом эндокринологическом обследовании в 11,5 года (табл. 4, рис. 3) выявлена задержка роста (SDS=-2,33) при своевременно начавшемся половом созревании (Таннер 2). КВ незначительно отставал от хронологического. В связи с низким уровнем ИФР-1 (SDS=-3,61) были проведены стимуляционные тесты с клофелином и инсулином — выявлена низкая пиковая секреция СТГ — максимально до 2,15 и 3,4 нг/мл соответственно, что подтвердило недостаточность СТГ. Данных за гипотиреоз и гипокортицизм не получено. При проведении МРТ гипофиза структурной патологии не выявлено, отмечены признаки перегрузки железом. В течение следующего года терапия рГР не была инициирована.

**Table:** Таблица 4. Показатели физического развития и результаты лабораторных исследований пациентки А.Table 4. Indicators of physical development and the results of laboratory studies of patient A. *3 мес терапии рГР.**1 год терапии рГР.***2 года 3 мес терапии рГР.

Возраст, лет	Рост, см (SDS)	Вес, кг (SDSимт)	Половой статус по Таннеру	Костный возраст, годы	ТТГ, мкМЕ/мл	свТ4, пмоль/л	Кортизол, нмоль/л	Пролактин, мкМЕ/мл	ИФР-1, нг/мл (SDS)	ЛГ, мМЕ/мл	ФСГ, мМЕ/мл	Эстрадиол, пмоль/л	Глюкоза плазмы натощак, ммоль/л
11,5	129,0 (-2,33)	30,3 (0,15)	В2Р2	11	5,5	13,4	326	531	74 (-3,61)	0,8	1,9	139	3,7
12,5	133,8 (-2,71)	30,8 (-0,61)	В2Р2	12	4,5	12,4	969	648	47 (-5,40)	2,5	3,2	100	-
13,0 *	135,5 (-2,93)	31,8 (-0,69)	В2Р2	12	3,9	13	375	148	275 (0,01)	1,2	1,6	175	5,0
13,8 **	142,5 (-2,54)	32,0 (-1,78)	В2Р2	13	3,7	14,5	384	489	184 (-1,55)	3,2	2,5	143	3,1
15,0 ***	147,4 (-2,32)	43,5 (0,05)	В3Р2	13	3,4	14,5	211	565	153 (-2,55)	3,6	5,7	91	5,6

**Figure:**
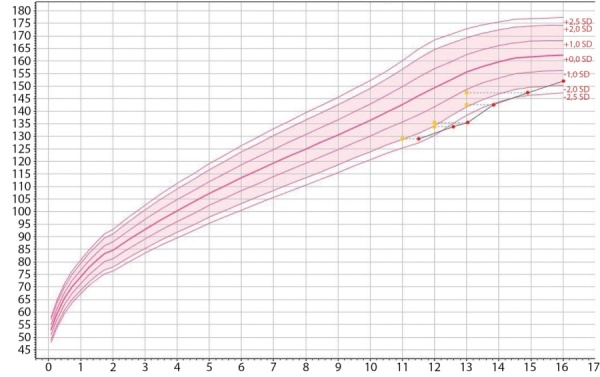
Рисунок 3. График роста пациентки А.Примечание к рисунку: по вертикали — рост, см; по горизонтали — возраст, годы; измерения роста в соответствии с хронологическим (красные точки) и костным (желтые точки) возрастом.Figure 3. Growth chart of patient A.

В 12,5 года (табл. 4, рис. 3) отмечено нарастание степени задержки роста (SDS = -2,71), половое развитие без динамики (Таннер 2). КВ, как при предыдущем обследовании, незначительно отставал от хронологического. Данных за нарушение секреции других питуитарных гормонов, кроме СТГ, не получено; уровень половых гормонов соответствовал началу пубертата; отмечено прогрессирующее снижение ИФР-1 (SDS=-5,40).

С 12,8 года инициирована терапия рГР в дозе 0,033 мг/кг/сут.

В 13,0 года на фоне лечения рГР (табл. 4, рис. 3) отмечена нормализация уровня ИРФ-1 (SDS=+0,01), остальные гормональные показатели в пределах возрастной нормы, КВ без динамики.

Отмечалось повышение гликированного гемоглобина (HbA1c) до 7,1%, что было расценено как возможная погрешность на фоне БТМ. По данным ПГТТ показатели гликемии в пределах нормы (табл. 5). Терапия рГР была продолжена.

В 13,8 года (табл. 4, рис. 3) на фоне терапии рГР отмечена положительная динамика: SDS роста =-2,54, скорость роста 8,7 см/год. В связи с отсутствием прогрессии полового развития (Таннер 2), низконормальным уровнем половых гормонов (табл. 4), проведен тест с аналогом гонадотропин-рилизинг-гормона, получен пубертатный уровень гонадотропинов (табл. 6).

В 15 лет случайно выявлено повышение глюкозы крови до 7,2 ммоль/л, пациентка была направлена на эндокринологическое обследование (табл. 4, рис. 3). Тенденция к нормализации роста сохранялась (SDS=-2,32), увеличилось отставание КВ отхронологического. Гормональные показатели без изменений, гликемия натощак в пределах нормы (табл. 4). При поведении ПГТТ гликемия натощак и через 2 ч после приема 75 г глюкозы в пределах нормы (табл. 5); инсулин 10 мкЕд/мл (N 3–27) и С-пептид 725 пмоль/л (N 260-1730).

**Table:** Таблица 5. Пероральные глюкозотолерантные тесты пациентки А. в 13 и 15 летTable 5. Oral glucose tolerance tests of patient A. at 13 and 15 years old

Возраст, лет	Глюкоза, ммоль/л
0 мин	60 мин	120 мин
13,0 лет	5,2	-	6,4
15,0 лет	5,6	9,5	7,3

**Table:** Таблица 6. Тесты с аналогом гонадотропин-рилизинг-гормона пациентки А. в 13,8 годаTable 6. Tests with a gonadotropin-releasing hormone analog in patient A. at 13.8 years

Гормоны/временная точка	0 ч	1 ч	4 ч
ЛГ, мМЕ/мл	3,4	9,4	16,8
ФСГ, мМЕ/мл	3,2	4,7	7,7

Пациентке был установлен НМГ на 5 дней (рис. 4), выявлены эпизодические подъемы гликемической кривой постпрандиального характера (максимальные значения 171 мг/дл (9,5 ммоль/л)). Средний уровень гликемии 6,1±0,8 ммоль/л.

**Figure:**
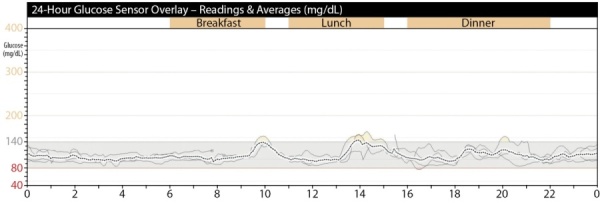
Рисунок 4. Результаты 5-дневного непрерывного мониторинга гликемии пациентки А.Примечание к рисунку: 140 мг/дл = 7,8 ммоль/л, 80 мг/дл = 4,5 ммоль/л.Figure 4. Results of 5-day continuous glycemic monitoring of patient A.

Диагноз и тактика ведения

Гипопитуитаризм (на момент наблюдения изолированная недостаточность СТГ) манифестировал у пациентки со вторичным гемохроматозом на фоне БТМ в 11 лет. На основании проведенного в 15 лет обследования был установлен диагноз нарушенная толерантность к глюкозе. По результатам мониторинга гликемии сахароснижающая терапия не была показана. Было рекомендовано соблюдение диеты с ограничением легкоусвояемых углеводов, продолжить терапию рГР. С учетом увеличения массы тела и низкого уровня ИРФ-1 скорректирована доза рГР.

Исход

Пациентка с недостаточностью СТГ и нарушенной толерантностью к глюкозе вследствие перегрузки железом на фоне трансфузионно-зависимой БТМ продолжает наблюдаться у эндокринолога по месту жительства. Со слов матери, девочка прекратила терапию рГР через 3 мес после описанного визита в 15 лет; в 16 лет рост девочки составил 152 см; патологические значения гликемии повторно не выявлялись; сахароснижающая терапия не назначалась; в связи с отсутствием прогрессирования полового созревания в 16 лет начата терапия валериатом эстрадиола.

## ОБСУЖДЕНИЕ

АДБ — это редкая форма врожденной аплазии кроветворения, развивающаяся в результате апоптоза предшественников эритроцитов в костном мозге, обусловленная мутацией одного из генов, кодирующих рибосомальные белки. Классический вариант АДБ выявляется у 7 из 1 млн живых новорожденных. Основными методами лечения на настоящий момент являются: регулярные трансфузии эритроцитарной массы, длительная глюкокортикоидная терапия или аллогенная трансплантация стволовых кроветворных клеток [2][4].

У пациентов с ДБА в половине случаев выявляются сопутствующие врожденные пороки развития черепа, верхних конечностей, сердца, мочеполовой системы, воспалительные изменения слизистой ротовой полости [4]. В литературе описаны сопутствующие АДБ эндокринные заболевания, обусловленные, по-видимому, как основным заболеванием, так и методами лечения (вторичная перегрузка железом, ятрогенный синдром Кушинга). Ввиду низкой встречаемости АДБ на момент написания статьи системные данные о сопутствующей эндокринной патологии ограничены [5][6].

По результатам анализа регистра пациентов с АБД в Северной Америке, включающего данные 57 человек (38 — с постоянными трансфузиями, 12 — глюкокортикоидзависимые и 7 — в ремиссии), 53% пациентов имели по крайней мере одно эндокринное осложнение, в т.ч. надпочечниковую недостаточность (32%), гипогонадизм (29%), гипотиреоз (14%), дефицит гормона роста (7%), сахарный диабет (2%), несахарный диабет (2%). Из 33 пациентов с доступными антропометрическими данными 10 имели задержку роста (SDS роста ниже -2,0). У 50% выявлялись дефицит или недостаточность витамина D. Все осложнения, кроме надпочечниковой недостаточности, чаще встречались у пациентов, постоянно нуждавшихся в трансфузиях донорской эритроцитарной массы [2]. Также на фоне перегрузки железом описано развитие гипопаратиреоза [7].

Согласно данному регистру, случай развития 4 сопутствующих эндокринных заболеваний у одного пациента с АДБ является редким. При поиске информации в базах PubMed, EMBASE, GoogleScholar не было найдено клинических случаев пациентовс АДБ и развившимися на фоне трансфузионной терапии нарушениями углеводного обмена.

Бета-талассемия — наиболее часто встречающаяся наследственная анемия, которая характеризуется нарушением синтеза бета-цепей гемоглобина. Клинически это нарушение проявляется анемией различной степени тяжести. Наиболее тяжелая форма —БТМ, с менее выраженными проявлениями — бета-талассемия интермедиа и минор (промежуточная и малая формы). Также возможны бессимптомные формы. Ежегодная встречаемость бета-талассемии, сопровождающейся клиническими проявлениями, в мире составляет около 1 на 100 000 человек, а в Европе — 1 на 10 000 человек [8].

Трансфузии эритроцитарной массы являются одним из основных методов лечения. Вследствие частых трансфузий гематологические пациенты со временем формируют осложнения постепенно нарастающей перегрузки железом: различные эндокринопатии, фиброз печени и функциональные нарушения сердца. Для выявления перегрузки железом проводятся исследование ферритина, биопсия печени, МРТ печени и сердца [8].

У пациентов с высокой концентрацией железа в печени при БТМ продемонстрирована высокая распространенность эндокринопатий, среди детей младше 12 лет она составила 23,3% [9]. К наиболее распространенным эндокринопатиям у детей сБТМ относятся гипотиреоз (9,2%), нарушения углеводного обмена (7,5%) и гипопаратиреоз (6,7%) [9]. В более старшем возрасте отмечена также высокая распространенность задержки роста (в зависимости от базы данных — от 8 до 14%), гипогонадизма (от 50 до 97,7%) и сахарного диабета (от 0 до 41%), с наибольшим числом случаев среди пациентов старшего возраста и высоким уровнем ферритина плазмы, причем статистически значимо большее число случаев зафиксировано упациентов, получающих гемотрансфузии [8].

Для пациентов с высокой концентрацией ферритина типичен наибольший риск эндокринных осложнений [9]. Так как организм не может освобождаться от железа самостоятельно, для его выведения с мочой и стулом используются хелаторы — препараты, связывающие железо. Применение хелаторов может быть ограничено в связи со сложностью введения парентеральных форм и выраженностью побочных эффектов [8]. Использование комбинированных схем хелатотерапии по сравнению смонотерапией приводит к значительному снижению распространенности эндокринопатий. Отмечено также, что на фоне терапии хелаторами железа отдельные эндокринопатии могут носить обратимый характер [9].

Таким образом, в описываемых нами клинических случаях у пациентов с перегрузкой железом на фоне трансфузионно-зависимых наследственных анемий выявлены наиболее типичные для этого состояния эндокринопатии. У обоих пациентов диагностированы недостаточность СТГ, нарушения полового созревания (гипогонадотропный гипогонадизм у пациента с АДБ и остановка полового созревания на стадии Таннер 2 у пациентки с БТМ) и нарушения углеводного обмена (сахарный диабет и нарушенная толерантность к глюкозе). У первого пациента также были зафиксированы вторичный гипотиреоз и гипокортицизм, самопроизвольно купировавшиеся при наблюдении, вероятно, вследствие проводимой хелаторной терапии.

## Задержка роста и дефицит гормона роста

Данные по лечению задержки роста при АДБ ограничиваются единственным опубликованным исследованием, в котором было проведено сравнение показателей роста 19 пациентов, получавших рГР, и 44 пациентов без лечения. Было продемонстрировано, что дети, которым назначалась терапия рГР, исходно имели выраженную задержку роста по сравнению с популяционными нормативами; в течение 4 лет на фоне лечения скорость роста значительно увеличивалась; и через 2 года терапии значение SDS роста статистически значимо не отличалось от других пациентов [10].

В связи с высокой распространенностью БТМ причины нарушений роста при этой анемии изучены достаточно хорошо. Считается, что задержка роста при БТМ имеет мультифакториальный генез — развивается вследствие влияния гипоксии на фоне хронической анемии, перегрузки железом гипофиза и токсичности хелаторной терапии. Но если вследствие первых двух причин формируется пропорциональная задержка роста, то побочным эффектом хелаторной терапии, в частности дефероксамина, является диспропорциональная туловищная низкорослость с платиспондилией (уплощение тел позвонков) и дисплазией длинных трубчатых костей [1][8].

В 2021 г. был проведен метаанализ, объединивший данные 74 исследований с 1978 по 2019 гг., по результатам которого у 41,1% пациентов с БТМ выявлено отставание роста от среднеродительского, у 48,9% — низкорослость в соответствии страдиционными критериями (SDS роста ниже -2,0), а у 26,6% — дефицит СТГ (определялся как пик СТГ в ходе стимуляционного теста ниже 5 нг/мл) [11].

Мнения о целесообразности назначения рост-стимулирующей терапии пациентам с БТМ и недостаточностью СТГ весьма неоднозначны. Так как у пациентов с задержкой роста выявляются относительно низкие уровни ИРФ-1, предполагается, что основной причиной снижения скорости роста является снижение производства ИРФ-1 печенью. В 2020 г. опубликован Кохрановский обзор, объединивший результаты исследований эффективности терапии рГР при БТМ с доказанной недостаточностью СТГ. Согласно литературным данным, на терапии рГР отмечается улучшение отдельных антропометрических показателей, в частности скорости роста, в течение первого года лечения, однако отсутствуют исследования, которые бы продемонстрировали влияние терапии на конечный рост пациентов [12].

Согласно клиническим рекомендациям по лечению эндокринопатий при БТМ, рекомендуемая доза рГР составляет 0,025–0,05 мг/кг. Принципы диагностики и наблюдения в период лечения не отличаются от других причин СТГ-недостаточности [1].

В описываемых клинических случаях пациенты получали рГР в дозе 0,033 мг/кг. На этом фоне отмечена нормализация значений ИРФ-1, но они оставались ниже среднепопуляционных для соответствующего возраста, пола и стадии полового созревания. Можно предположить, что небольшой ростовой эффект терапии отчасти обусловлен этим фактором, и титрация дозы рГР в пределах рекомендуемого диапазона под контролем уровня ИРФ-1 могла бы способствовать большей прибавке роста. В то же время необходимо отметить, что к 18-летию рост пациента с АДБ достиг нижней границы популяционной нормы, и с учетом открытых зон роста и терапии рГР ожидается, что пациент продолжит расти. У девочки с БТМ к 16 годам рост нафоне терапии рГР также достиг нормальных значений.

## Гипотиреоз

Аналогично другим эндокринопатиям, наличие гипотиреоза зависит от наличия гемохроматоза. Согласно метаанализу 2021 г., распространенность тотального гипотиреоза у пациентов, зависимых от трансфузий, составляет 16,22%, у независимых — 7,22% [13]. Интересно, что по данным Малазийского исследования, где распространенность гипотиреоза у пациентов с БТМ составила 21,6%, более чем в половине случаев был подтвержден центральный гипотиреоз [14].

У пациента в первом клиническом случае в период наблюдения был выявлен центральный гипотиреоз, по поводу чего была назначена терапия левотироксином. Однако после самостоятельной отмены терапии пациентом в 17,9 лет диагноз не подтвердился.

## Гипопаратиреоз

В исследовании, прицельно изучавшем нарушения кальций-фосфорного обмена у детей с БТМ, первичный гипопаратиреоз был выявлен у 3,4%, субклинический — у 52,3%, а вторичный — у 34% [15]. Считается, что причиной гипопаратиреоза может быть как перегрузка паращитовидных желез железом, так и подавление секреции паратгормона вследствие повышенной резорбции костной ткани при активном гемопоэзе на фоне хронической анемии [1]. В описываемых клинических случаях нарушений кальций-фосфорного обмена не выявлено.

## Гипогонадизм

Распространенность гипогонадизма составляет 27,6% [16]. Обычно развивается центральный гипогонадизм вследствие перегрузки железом гипофиза. Гипогонадизм может манифестировать в любом возрасте: как задержка пубертата или остановка полового созревания у подростка с самостоятельной инициацией пубертата, а также как вторичная аменорея или снижение либидо и эректильная дисфункция во взрослом возрасте. Необходимо учитывать, что гипогонадизм повышает риск остеопороза [17].

Нарушения полового созревания у описываемых пациентов манифестировали как задержка полового созревания. После подтверждения диагноза пациент с АДБ начал получать препарат эфиров тестостерона в стандартных для периода инициации полового созревания дозах. В противоположность этому, у пациентки с БТМ отмечен пубертатный подъем ЛГ в ходе теста с аналогом гонадотропин-рилизинг-гормона, что предполагало дальнейшее самостоятельное половое созревание. Однако, судяпо тому, что в 16 лет пациентке была назначена терапия валериатом эстрадиола, можно говорить об остановке полового созревания.

## Нарушения углеводного обмена

Диабет, ассоциированный с гемохроматозом, развивается в результате сочетания нарушения секреции инсулина пораженными бета-клетками и формирования инсулинорезистентности при перегрузке железом мягких тканей [18].

По данным метаанализа 2019 г., включавшего данные 44 ранее опубликованных исследований, распространенность СД среди пациентов с БТМ составила 6,5% (с максимальным показателем 7,5% на Ближнем Востоке), нарушенной гликемии натощак — 17,2%, нарушенной толерантности к глюкозе — 12,5% [18].

С возрастом отмечается увеличение распространенности и степени тяжести нарушений углеводного обмена. При обследовании взрослых пациентов с недавно выявленным СД обращает на себя внимание более ранняя манифестация СД у женщин по сравнению с мужчинами, инсулинорезистентность и частое сочетание с другими осложнениями гемохроматоза. Отмечено также, что нарушения углеводного обмена при гемохроматозе у детей чаще и более тяжело проявляются при более выраженной перегрузке железом и при дефиците цинка [18][19].

Для оценки состояния углеводного обмена у пациентов с БТМ рекомендуется вычислять индекс HOMA, однако существенным недостатком данного метода является отсутствие референсных значений для пациентов с БТМ. Также каждые 6 мес рекомендуется проводить исследование цинка [20].

Критерии диагностики нарушений углеводного обмена при гемохроматозе не отличаются от таковых у пациентов без гематологических заболеваний, однако до сих пор остается дискутабельным, какой метод подходит для скрининга [3][20]. Например, международные клинические рекомендации, кроме исследования гликемии каждые 6 мес, рекомендуют проводить ПГТТ в 10, 12, 14 и 16 лет, а затем ежегодно [1], либо ежегодно, начиная с 10 лет [18]. Но даже при ежегодном проведении ПГТТ можно пропустить нарушения углеводного обмена, которые могут проявляться постпрандиальными гипергликемиями в домашних условиях, в частности, у ряда пациентов фиксируются гипергликемии в течение 2 ч после приема глюкозы. В связи с этим использование систем НМГ является более показательным [20].

Выбор критерия компенсации углеводного обмена при сахарном диабете и хронической анемии также затруднен. Как отмечено в клинических рекомендациях, уровень HbA1c является «слабым» маркером. Рекомендуется рассматривать в качестве альтернативного метода измерение фруктозамина, который отражает показатели гликемии за предшествующие 2–3 нед [20].

У описываемых пациентов впервые нарушение углеводного обмена было диагностировано на основании случайного выявления гипергликемии натощак, что демонстрирует достаточную эффективность этого исследования в качестве скринингового. В то же время стандартный ПГТТ оказался полезен лишь для выявления сахарного диабета у пациента с АДБ. Но при преддиабете, как продемонстрировано в случае с пациенткой с БТМ, ПГТТ не совсем отражает состояние углеводного обмена. У данной пациентки нарушенная толерантность к глюкозе была диагностирована по данным НМГ (подъемы гликемии до 9,5 ммоль/л), хотя в ходе ПГТТ гликемия натощак и через 2 ч после еды соответствовала нормальным значениям.

Уровень инсулина у пациентов свидетельствовал об инсулинорезистентности, что типично для нарушений углеводного обмена на фоне гемохроматоза. Исследование HbA1c нами не проводилось в связи с высокой трансфузионной зависимостью пациентов (эритромасса вводится ежемесячно). Позволит ли уровень фруктозамина адекватно оценить компенсацию углеводного обмена у подобных пациентов, вызывает сомнения, так как для данного показателя отсутствуют стандартизированные целевые значения.

Клинические рекомендации по лечению эндокринопатий при бета-талассемии

В 2013 г. были приняты международные Клинические рекомендации по лечению эндокринопатий при бета-талассемии. Рекомендуется каждые 6–12 мес проводить антропометрические измерения: при первом обследовании записывать рост родителей и рассчитывать среднеродительский рост; на каждом визите измерять рост стоя и сидя, массу тела и окружность головы (первые 2 года жизни); рассчитывать скорость роста за год, индекс массы тела и соотношение верхний/нижний сегмент; оценивать показатели роста по этническим нормативам или нормативам Всемирной организации здравоохранения; оценивать половой статус по Таннеру [1].

Задержку роста рекомендуется диагностировать не только при выявлении показателя линейного роста ниже 3-го центиля или -2SD, но и при снижении скорости роста за 6–12 мес ниже 25-го центиля, а также при отставании роста отсреднеродительского [1].

Помимо роста и полового статуса, необходимо ежегодно оценивать следующие показатели:

## ЗАКЛЮЧЕНИЕ

Представленные клинические случаи демонстрируют развитие наиболее распространенных эндокринных осложнений у пациентов с трансфузионно-зависимыми анемиями — нарушения роста, полового созревания и углеводного обмена. Это подчеркивает необходимость регулярного эндокринологического обследования подобных пациентов для оценки антропометрических, биохимических, гормональных и рентгенологических показателей. Нарушения функции желез внутренней секреции могут носить обратимый характер на фоне хелаторной терапии. Своевременное выявление недостаточности СТГ и назначение терапии позволяют достигать нормальных показателей конечного роста. Нарушения полового созревания требуют наблюдения и дифференциальной диагностики с конституциональной задержкой полового созревания. С целью выявления нарушений углеводного обмена рекомендуется проводить исследование гликемии натощак, а при выявлении пограничных или патологических значений — ПГТТ и НМГ; исследование гликированного гемоглобина нецелесообразно.

## ДОПОЛНИТЕЛЬНАЯ ИНФОРМАЦИЯ

Источники финансирования. Работа выполнена по инициативе авторов без привлечения финансирования.

Конфликт интересов. Авторы декларируют отсутствие явных и потенциальных конфликтов интересов, связанных с содержанием настоящей статьи».

Участие авторов. Витебская А.В. — концепция и дизайн исследования, ведение пациентов, написание статьи; Бугакова Е.С. — ведение пациентов, подготовка обзора литературы и описания клинического случая; Писарева Е.А. — ведение пациентов, подготовка описания клинического случая; Тихонович Ю.В. — ведение пациентов, внесение правки. Все авторы одобрили финальную версию статьи перед публикацией, выразили согласие нести ответственность за все аспекты работы, подразумевающую надлежащее изучение и решение вопросов, связанных с точностью или добросовестностью любой части работы.

Согласие пациента. Пациенты добровольно подписали информированные согласия на публикацию персональной медицинской информации в обезличенной форме в журнале «Проблемы эндокринологии».
